# The relationships between patient safety culture and sentinel events among hospitals in Saudi Arabia: a national descriptive study

**DOI:** 10.1186/s12913-023-09205-0

**Published:** 2023-03-18

**Authors:** Samar Binkheder, Yasser A. Alaska, Alia Albaharnah, Rawan Khalid AlSultan, Nawaf Mubarak Alqahtani, Anas Ahmad Amr, Nawfal Aljerian, Rabab Alkutbe

**Affiliations:** 1 Medical Informatics and E-Learning Unit, Medical Education Department, College of Medicine, King Saud University, Riyadh, 12372 Saudi Arabia; 2 Technical Affairs, Saudi Patient Safety Center (SPSC), Riyadh, 12264 Saudi Arabia; 3 Emergency Medicine, College of Medicine, King Saud University, Riyadh, 12372 Saudi Arabia; 4 Saudi Critical Care Society, Riyadh, 12243 Saudi Arabia; 5 Department of Emergency Medical Services, King Saud Bin Abdulaziz University for Health Sciences (KSAU-HS), Riyadh, 14611 Saudi Arabia; 6 Medical Referrals Center, Ministry of Health, Riyadh, Saudi Arabia

**Keywords:** Patient safety culture, Sentinel events, Adverse events, Hospitals, Descriptive study, Correlation analysis, Saudi Arabia

## Abstract

**Background:**

Sentinel events (SEs) can result in severe and unwanted outcomes. To minimize the fear of sentinel events reporting and the occurrence of sentinel events, patient safety culture improvements within healthcare organizations is needed. To our knowledge, limited studies explored the relationships between patient safety culture and sentinel events on a local level and no research has been conducted at the national level in Saudi Arabia.

**Objectives:**

This study aimed to explore the relationships between the patient safety culture and the reported-SEs on a national level during the year 2020 in Saudi hospitals.

**Methods:**

This was a descriptive study. We utilized two data sources (the reported-SEs and the patient safety culture survey) that were linked using hospitals information. To explore the relationships between patient safety culture and reported-SEs rates, we performed descriptive statistics, a test of independence, post-hoc analysis, correlation analysis, and multivariate regression and stepwise analyses.

**Results:**

The highest positive domain scores in patient safety culture domains in the Saudi hospitals (*n* = 366) were “Teamwork Within Units” (80.65%) and “Organizational learning-continuous improvement” (80.33%), and the lowest were “Staffing” (32.10%) and “Nonpunitive Response to Error” (26.19%). The highest numbers of reported-SEs in 103 hospitals were related to the contributory factors of “Communication and Information” (63.20%) and “Staff Competency and Performance” (61.04%). The correlation analysis performed on 89 Saudi hospitals showed that higher positive patient safety culture scores were significantly associated with lower rates of reported-SEs in 3 out of the 12 domains, which are “Teamwork Within Units”, “Communication Openness”, and “Handoffs and Transitions”. Multivariate analyses showed that “Handoffs and Transitions”, “Nonpunitive Response to Error”, and “Teamwork Within Units” domains were significant predictors of the number of SEs. The "Staff Competency and Performance" and "Environmental Factors" were the most contributory factors of SEs in the number of significant correlations with the patient safety culture domains.

**Conclusion:**

This study identified patient safety culture areas of improvement where hospitals in Saudi Arabia need actions. Our study confirms that a more positive patient safety culture is associated with lower occurrence of sentinel events. To minimize the fear of sentinel events reporting and to improve overall patient safety a culture change is needed by promoting a blame-free culture and improving teamwork, handoffs, and communication openness.

**Supplementary Information:**

The online version contains supplementary material available at 10.1186/s12913-023-09205-0.

## Background

Medical errors were reported as the third major cause of death in the United States (US) in 2016 and it has been estimated that avoidable errors contribute to 1708 death per year in the United Kingdom [1–3]. Medical errors can result in financial costs estimated at billions of dollars yearly as well as psychological and physical harm to patients leading to a lack of trust in the healthcare systems [4]. To *Err Is Human* report [5] was published by the Institute of Medicine (IOM) in 2000 and highlights the responsibility of healthcare professionals in improving the patient safety culture aiming for better patient outcomes [6]. This problem is not blamed on people but rather on healthcare systems that need to be safer [5]. The most severe errors are called sentinel event (SE), which is defined by the Joint Commission as “the patient safety event that results in death, permanent harm, or severe temporary harm” [7]. Furthermore, the Saudi Patient Safety Center explains SE as “an adverse event in health care delivery or other services, which either leads to or has the potential to lead to catastrophic outcomes” [8]. In Saudi Arabia, the number of complaints and claims relevant to adverse events against healthcare providers has increased [9]. During the years from 2016 to 2019, there were 727 SEs reported in Saudi Arabia where the top SEs were 38.4% unexpected patient death, 19.4% maternal death, and 11.7% unexpected loss of limb of function [10]. Thus, the reduction of SEs and harm to patients is one of the groundwork of patient safety that needs to be continuously improved. Therefore, the Saudi Patient Safety Center [11] was established in 2017 as one of Saudi’s National Transformation Vision of 2030 initiatives to improve patient safety in a national level in Saudi Arabia.

There are several actions to be taken in the case of suspected or confirmed SEs at hospital-level, including a reporting system that helps to identify a root cause analysis, followed by a corrective action plan [12]. Although reporting medical errors is mandated in many healthcare systems, in the US, for example, only 10% of the events are reported [13]. According to Anderson and Abrahamson (2017), 15% of the hospitals that have reported errors had a corrective action plan that involved system changes [14]. An observational study conducted by Hamilton et al. (2017) to evaluate the variance reporting systems, found that during six weeks five observed adverse events were not reported by any reporting systems whereas only two adverse events were handwritten reported [15]. A scoping review of patient safety in Saudi hospitals found that there is evidence of some cultural aspects that might negatively influence patient safety such as the tendency to report errors with no harm more than those with harm, punitive response to error, and a need for confidential incident reporting [16]. These findings in agreement with other studies suggested that adverse events require a culture change to minimize the fear of reporting [15, 17]. Therefore, one of the critical processes is assessing areas of weaknesses and strengths in patient safety culture among healthcare organizations, including the reporting culture, to maintain better healthcare outcomes and safer organizations.

Patient safety culture (PSC) is one of the vital elements that is evident to affect the patient safety level within healthcare organizations. A systematic review found that the key gaps in patient safety culture in Saudi Arabia are ineffective leadership, a blame culture, inadequate staffing, and poor communication. On the other hand, they identified the s patient safety culture strength factors which are supportive organizational attitudes to continuous improvement, good teamwork within units, and support from management [9]. Some studies that investigated the impact of PSC on reporting events agreed that positive culture improves patient safety standards in collaborating with the capacity and fearlessness to report errors [18, 19]. For instance, Mardon et al. [20] hypothesized that higher scores on the PSC survey were associated with lower rates of adverse events. Among the top factors that contributed to underreporting of SEs and lower PSC in Saudi Arabia and worldwide are blame culture, poor leadership, lack of staffing, poor communication, fear of reporting sequel, human and work environment factors, the reporting system used, compliance with policy and procedure, fear of punitive action, and lack of understanding of patient safety events [16, 21–27]. A survey conducted by the Netherlands Federation of University Medical Centers (2020) to assess the handling and learning from SEs found that 5 out of 8 hospitals reported that the reoccurrence of similar events was caused by culture and communication [28]. Similarly, a study conducted in Saudi Arabia to investigate the most common SEs reported by the hospitals found that policies and procedures, ineffective communication, shortage of staff, and lack of competencies are the common causes [10]. Therefore, overcoming the barriers and developing a non-blaming, non-punitive learning culture may have a significant role in the reporting system and enhance patient safety initiatives [29].

Despite the challenges of reporting the SEs that face the health systems, monitoring and analyzing these reports significantly contributes to the effectiveness of improving patient safety [12]. A study assessed PSC and included 13 hospitals located in Riyadh city in Saudi Arabia found that underreporting of errors due to the fear of blame culture even when harm occurs can result in threatening patient safety and neglecting valuable information on errors as well as inability to analyze these errors [30]. PSC is a crucial part of organizational culture in which healthcare providers recognize patient safety performance as the highest priority measure to prevent patient harm [31]. Data generated from PSC is multi-dimensional and can be used to explore relationships and correlations among their attributes and linked to patients’ outcomes; however, there is a gap in the literature due to limited studies in this context [32]. More specifically, linking the aggregated PSC data with the occurrence of SEs and their contributory factors might have an impact on identifying cultural factors that can positively or negatively influence patient safety within healthcare systems and processes. A systematic review aimed to identify connections between PSC and patients’ outcomes found that there is evidence of relationships between them, such as the negative correlations between PSC and mortality rates [6]. We hypothesized that hospitals with a more positive PSC have lower numbers of SEs. Furthermore, this study can contribute to providing nationwide hospital-level aggregated PSC and reported-SEs results that can help healthcare leaders and decision-makers to gain insights about areas that need improvements. To our knowledge, limited studies explored the relationships between patient safety culture and sentinel events on a local level and no research has been conducted at the national level in Saudi Arabia.

## Objectives

This study aimed to describe and explore the relationships between the PSC and the sentinel events (number of reported SEs, the categories of contributory factors, and the level of harm) by linking the two data sources during the year 2020 in Saudi hospitals.

## Methods

### Study design

This was a descriptive and exploratory study. Ethical approval was obtained by the institutional review board at King Saud Medical City (IRB Project No. E-22–6551).

### Study setting and data sources

The study utilized secondary data from two data sources collected at a national level in Saudi Arabia at the Saudi Patient Safety Center, which are the patient saftey culture survey and the sentinel events reports. The hospitals included all healthcare sectors, which are the ministry of health (MOH), government non-MOH, and private. 

#### Dataset 1: The patient safety culture survey

The Hospital Survey on Patient Safety Culture (HSPSC) questionnaire is a tool developed by the Agency for Healthcare Research and Quality (AHRQ) [33] to evaluate the PSC [34]. The survey is distributed yearly through the Saudi Patient Safety Center targeting all healthcare workers anonymously in Saudi hospitals in both Arabic and English languages [35]. Hospitals were required to register their interest in participating with the Saudi Patient Safety Center by creating an account on the designated electronic survey platform through the authorized point of contact who is an authorized staff at each hospital. The point of contact updated the facility and staff information such as the number of beds, and the number of staff per department. After that, the surveys were distributed electronically to the hospitals by unique links. Surveys were filled in real-time with the ability to visualize the progress by the Saudi Patient Safety Center team, governance sectors, and hospitals. The maximum number of filled surveys was determined based on the total number of staff for each hospital as self-reported by the hospital, where the system did not allow extra surveys beyond the total number of staff. After closing the 8-week cycle of data collection, the hospitals that did not meet the Saudi Patient Safety Center criteria as the following: non-verified registration, non-completed registration (at the end of each cycle), and hospitals with less than 10 completed surveys were excluded.

We utilized the HSPSC survey responses that were collected by the Saudi Patient Safety Center during the period from January 2021 and March 2021. The final dataset included 124,891 responses from 366 hospitals. The average hospital response rate during this cycle was 63.81% (ranging from 2 to 100%). We removed duplicated records and calculated the PSC domains according to the AHRQ guidelines that facilitate comparison between hospitals and the entire database per item, per domain, and per hospital’s domains. The used version in this cycle was version 1 of the HSPSC, which consists of 42 items measuring 12 PSC domains, which are (D1) teamwork within units, (D2) supervisor/manager expectations and actions promoting patient safety, (D3) organizational learning—continuous improvement, (D4) management support for patient safety, (D5) overall perceptions of patient safety, (D6) feedback and communication about error, (D7) communication openness, (D8) frequency of events reported, (D9) teamwork across units, (D10) staffing, (D11) handoffs and transitions, and (D12) nonpunitive response to error. The scoring of the items was a five-point Likert scale of agreement (1 = ‘strongly disagree’ to 5 = ‘strongly agree’) or a scale of frequency (1 = ‘never’ to 5 = ‘always’). Furthermore, two additional PSC measures were included in our study, the patient overall safety grade (POSG) and the positive events reported (PER). For the calculation of the percent positive per hospital for these PSC measures, we counted “Excellent” and “Very Good” as positive responses for “Please give your work area/unit in this hospital an overall grade on patient safety” and counted “1 to 2 event reports”, “3 to 5 event reports”, and “6 to 10 event reports” as positive responses for “In the past 12 months, how many event reports have you filled out and submitted”. In addition, demographic and hospital data were also collected including region, bed capacity, staff position, work area, weekly working hours, and interaction with patients.

#### Dataset 2: The sentinel events reports

The Saudi Patient Safety Center provides a framework to standardize the root cause analysis process and direct the healthcare facility to improvement. The reporting healthcare facility should complete and submit the root cause analysis and a corrective action plan within 45 days of the SE. The nine categories of contributory factors (CF) (Process Issues, Human Factors, Equipment / Technology, Environmental Factors, Staff Competency and Performance, Manpower Planning Issues, Leadership and Safety Culture, Communication and Information, and Others) were based on the root cause analysis template [8]. Each category repeatedly asks a series of triggering questions and a checklist of sub-CFs until the root systemic causal factors that culminated in the SE are identified as described in the Saudi healthcare sentinel event manual [8]. The Saudi Patient Safety Center received the SE reports from healthcare providers and reviewed them internally according to the Saudi healthcare sentinel events manual criteria [8], including the root cause analyses that were reported by the hospitals for each event and the corrective action plan. Afterward, all data were manually entered after cleaning and reviewed on an excel sheet. All reports with no personal identifiers from January 2020 to December 2020 were included. The number of reported-SEs was 231 from 103 hospitals. The variables included in this study were event location, who was affected (patient, staff, or organization), the level of harm (death, permanent harm, severe temporary harm, no harm) or event outcome, and the categories of CFs.

### Data analysis

Descriptive statistics were performed by using measures of frequency to calculate frequencies and percentages [36] for both data sources (the PSC and the reported-SEs). All the PSC measures were calculated based on the hospital-level positive percentages and then were aggregated for all variables by calculating their averages. To assemble the final dataset, we merged the two data sources by matching hospitals to assess the relationships between the reported-SEs and PSC measures. The test of independence, F-test for numeric variables, was used to test if variables were dependent on the bed size of the hospitals and we reported the following: mean, standard deviation, F-test, and p-value. In addition to the F-test, the Tukey post-hoc test was used for pairwise comparisons between bed size groups ("50–100 beds", "101–200 beds", "201–300 beds", "301–500 beds", and "501 + beds") among all variables with significant differences. Bivariate analysis using spearman correlation was used to measure the relationships between the following variables: the number of reported-SEs, CFs, level of harm, and the PSC domains and measures. We visualized these correlations using a heatmap for all the variables. Multivariate regression was used to identify statistically significant PSC domain predictors for each of the following dependent variables: the number of sentinel events, percent positive of overall patient safety grade, and percent positive of events reported. Multivariate regression was also performed using a stepwise algorithm bidirectional selection [37]. R [38], Tableau [39], SPSS [40], and Microsoft Excel [41] were used for data analysis and visualization. The following R functions and packages were used: “vtable” [42], “TukeyHSD” [43], “cor” [44], “corrplot” [45], “ggcorrplot” [46], and “stepAIC” [47]. *P*-value < 0.05 was considered to be significant.

## Results

### Descriptive summary of the two data sources

Table 1 shows the percentages of reported-SEs based on the CFs and the positive percentages results of the PSC survey for the whole dataset. The highest CFs were “Communication and Information” (63.20%) and “Staff Competency and Performance” (61.04%). The reported-SEs that were categorized in either of these CFs were received from 75.73% of the reporting hospitals (Table 1). “Others” (11.26%) was the lowest CF which includes the unavailability of some policies, procedures, or resources. For the PSC survey (Table 1), the three highest-scored domains within the database were: “Teamwork Within Units” (80.65%), “Organizational learning-continuous improvement” (80.33%), and “Feedback and Communication About Errors” (65.31%). On the other hand, the three lowest-scored domains in the database were: “Communication Openness” (53.43%), “Staffing” (32.10%), and “Nonpunitive Response to Error” (26.19%). The database's average percentage of positive responses across 12 domains was 58.38%.

**Table 1:** A summary of the average percent positive responses of the patient safety culture (PSC) across 366 hospitals and the reported sentinel events (SEs) based on the category of contributory factors

**Patient Safety Culture (PSC) Survey**		
**Patient Safety Measure**	**Average of Hospital-level Percent Positive**	**AHRQ 2018** [48]
Teamwork Within Units	80.65%	82%
Organizational Learning—Continuous Improvement	80.33%	72%
Feedback & Communication About Error	65.31%	69%
Supervisor/Manager Expectations & Actions Promoting Patient Safety	64.39%	80%
Management Support for Patient Safety	64.10%	72%
Teamwork Across Units	59.92%	62%
Frequency of Events Reported	59.45%	67%
Overall Perceptions of Patient Safety	59.17%	66%
Handoffs & Transitions	55.50%	48%
Communication Openness	53.43%	66%
Staffing	32.10%	53%
Nonpunitive Response to Error	26.19%	47%
Across 12 Domains	58.38%	65%
Patient overall safety grade (POSG)	75.71%	78%
Positive events reported (PER)	50.00%	45%
**Reported Sentinel Events**		
**Category of Contributory Factor (CF)**	**Reported Sentinel Events (*n* = 231)**	**Hospitals Reported Sentinel Events (*n* = 103)**
Communication and Information	146 (63.2%)	78 (75.73%)
Staff Competency and Performance	141 (61.04%)	78 (75.73%)
Process Issues	137 (59.31%)	77 (74.76%)
Human Factors	131 (56.71%)	74 (71.84%)
Manpower Planning Issues	108 (46.75%)	73 (70.87%)
Leadership and Safety Culture	89 (38.53%)	59 (57.28%)
Equipment / Technology	58 (25.11%)	44 (42.72%)
Environmental Factors	36 (15.58%)	28 (27.18%)
Others	26 (11.26%)	25 (24.27%)
Not Categorized	35 (15.15%)	27 (26.21%)

The total number of SEs reported during 2020 was 231 from 103 hospitals across Saudi Arabia. The highest percentage of SEs (31.60%, *n* = 73) was reported in Riyadh region followed by Makkah region (22.94%, *n* = 53). The number of reported-SEs in our dataset was the highest (29%, *n* = 67) reported from 25 hospitals with a bed size of “301–500 beds”. For the PSC survey, the total number of respondents was 124,891 from 366 hospitals across different regions in Saudi Arabia. The PSC results showed that 22.92% (*n* = 28,622) of respondents were from hospitals with a bed size of “301–500 beds” (9.56%, *n* = 35). In addition, hospitals with a bed size of “50–100 beds” were the highest (54.37%, *n* = 199) with 25,403 (20.34%) respondents. The highest number of respondents were working in the emergency department (8.79%, *n* = 10,979). The highest number of respondents were registered nurses (34.02%, *n* = 42,485). Appendix 1 and  2 show a descriptive summary of the reported-SEs and the PSC survey before merging the two datasets.

### Linking the patient safety culture and the reported sentinel events

Table 2 shows the PSC measures (average positive events reported, average patient overall safety grade, and average percent positive across 12 PSC domains) linked to the hospitals with reported-SEs. After merging the PSC dataset with the sentinel events dataset, the number of reported-SEs was 195 which occurred in 89 hospitals. The highest number of SEs was found in “301–500 beds” bed size with 60 SEs (30.77%), which were reported from 21 different hospitals (23.6%). For the CFs, “Communication and Information” was the highest reported category (*n* = 128, 65.64%), followed by “Staff Competency and Performance” (*n* = 122, 62.56%), then “Process Issues” (*n* = 121, 62.05%). Among the 195 reported-SEs, 132 (67.69%) of the cases had resulted in death, and 13 (6.67%) of the cases resulted in permanent harm.

**Table 2:** A descriptive summary after linking the patient safety culture (PSC) measures to the reported sentinel events variables

Variable	Sentinel Events		Percent Positive Response of Patient Safety Culture (PSC) Measures		
	**Number of Reported Sentinel Events (%)**	**Number of Hospitals (%)**	**Positive Events Reported (PER)**	**Patient Overall Safety Grade (POSG)**	**Across 12 Domains**
**Bed Size**					
**50–100 beds**	23 (11.79%)	20 (22.47%)	53.14%	74.12%	56.84%
**101–200 beds**	42 (21.54%)	23 (25.84%)	50.78%	75.17%	56.57%
**201–300 beds**	25 (12.82%)	14 (15.73%)	48.83%	73.83%	54.70%
**301–500 beds**	60 (30.77%)	21 (23.6%)	48.24%	74.08%	53.09%
**501 + beds**	45 (23.08%)	11 (12.36%)	53.70%	73.90%	56.32%
**Category of Contributory Factor**					
**CF1: Communication and Information**	128 (65.64%)	70 (78.65%)	50.66%	74.31%	55.28%
**CF2: Staff Competency and Performance**	122 (62.56%)	70 (78.65%)	50.22%	73.84%	54.96%
**CF3: Process Issues**	121 (62.05%)	70 (78.65%)	50.44%	73.68%	55.14%
**CF4: Human Factors**	115 (58.97%)	66 (74.16%)	50.74%	73.75%	55.04%
**CF5: Manpower Planning Issues**	98 (50.26%)	69 (77.53%)	50.56%	74.02%	55.28%
**CF6: Leadership and Safety Culture**	83 (42.56%)	55 (61.8%)	50.13%	74.06%	55.05%
**CF7: Equipment / Technology**	47 (24.1%)	38 (42.7%)	49.83%	73.72%	54.58%
**CF8: Environmental Factors**	31 (15.9%)	24 (26.97%)	50.11%	70.98%	53.56%
**CF9: Others**	23 (11.79%)	22 (24.72%)	49.01%	72.24%	53.70%
**CF10: Not Categorized**	28 (14.36%)	21 (23.6%)	54.08%	75.91%	56.98%
**Level of Harm**					
**Death**	132 (67.69%)	72 (80.90%)	49.89%	73.65	54.83%
**Permanent Harm**	13 (6.67%)	10 (11.24%)	47.16%	73.49	52.59%
**Severe temporary harm**	26 (13.33%)	21 (23.60%)	53.75%	76.34	58.17%
**No Harm**	24 (12.31%)	17 (19.10%)	51.55%	74.78	55.47%
**Total**					
**Grand Total**	195 (100%)	89 (100%)	50.77%	74.31%	55.49%

Table 3 shows the results of test of independence based on bed size for each of the following: number of reported-SEs, category of CFs, level of harm, and PSC domains. There was a statistical significance difference among hospitals according to bed size for all reported-SEs and CFs, except for “Equipment/ Technology”, “Environmental Factors”, and “Others”. Using post-hoc analysis, we found that the difference in the reported-SEs rates was between “501 + beds” with each of “50–100 beds” (*p* = 0.0011),“101–200 beds” (*p* = 0.017), and “201–300 beds” (*p* = 0.034) as well as between “50–100 beds” with “301–500 beds” (*p* = 0.048). Furthermore, “Communication and Information” showed a difference between “301–500 beds” with “50–100 beds” (*p* = 0.017) and with “101–200 beds” (*p* = 0.025). The “Process Issues” showed that the difference was between “501 + beds” with “50–100 beds” (*p* = 0.004), “101–200 beds” (*p* = 0.004), and “201–300 beds” (*p* = 0.029). The “Human Factors” showed that the difference was between “501 + beds” with “50–100 beds” (*p* = 0.041) and with “101–200 beds” (*p* = 0.049). The level of harm showed a statistical significance difference only in “Death” (*p* = 0.0003). Among hospitals according to bed size and using post-hoc analysis the difference in “Death” was between “501 + beds” with each of “50–100 beds” (*p* = 0.0007), “101–200 beds” (*p* = 0.0009), and “201–300 beds” (*p* = 0.004). As for PSC domains, the results showed a statistical significance difference among hospitals according to bed size in 4 domains. The post-hoc analysis showed that the difference in “Teamwork Within Units” domain was between “301–500 beds” with “50–100 beds” (*p* = 0.0001) and with “101–200 beds” (*p* = 0.032). The difference for “Organizational Learning—Continuous Improvement” domain was between “50–100 beds” with “301–500 beds” (*p* = 0.02). The difference for “Communication Openness” domain was between “50–100 beds” and “301–500 beds” (*p* = 0.05). Lastly, “Handoffs and Transitions” domain showed a difference between “50–100 beds” and “301–500 beds” (*p* = 0.03).

**Table 3:** The relationship between bed size and the number of sentinel events, category of contributory factors, level of harm, and patient safety culture measures

Bed Size	50–100 beds	101–200 beds	201–300 beds	301–500 beds	501 + beds	F-Test	*p*-value
Variable	**N (Mean ± SD)**	**N (Mean ± SD)**	**N (Mean ± SD)**	**N (Mean ± SD)**	**N (Mean ± SD)**		
**Sentinel Events**							
**All Sentinel Events**	20 (1.15 ± 0.37) c,d	23 (1.83 ± 1.15) a	14 (1.79 ± 1.19) b	21 (2.86 ± 2.29) d	11 (4.09 ± 4.06) a, b, c	***F*** = 4.996**	0.001
**Category of Contributory factors**							
**CF1: Communication and Information**	15 (1.2 ± 0.41) a	21 (1.38 ± 0.74) b	10 (1.6 ± 0.84)	14 (2.79 ± 1.76) a, b	10 (2.6 ± 2.46)	***F*** = 4.177**	0.005
**CF2: Staff Competency and Performance**	12 (1.17 ± 0.39)	20 (1.35 ± 0.67)	11 (1.27 ± 0.65)	17 (2.41 ± 1.54)	10 (2.6 ± 2.46)	***F*** = 3.717**	0.009
**CF3: Process Issues**	12 (1.08 ± 0.29) a	21 (1.29 ± 0.64) b	10 (1.4 ± 0.7) c	18 (2.11 ± 1.18)	9 (3.22 ± 3.07) a,b,c	***F*** = 4.735**	0.002
**CF4: Human Factors**	13 (1 ± 0) a	18 (1.17 ± 0.38) b	11 (1.45 ± 0.82)	15 (2.53 ± 1.81)	9 (3 ± 3.54) a, b	***F*** = 3.706**	0.009
** CF5: Manpower Planning Issues**	15 (1 ± 0) a	19 (1.37 ± 0.68)	11 (1.27 ± 0.65)	16 (1.75 ± 0.93) a	8 (1.88 ± 0.99)	***F*** = 3.12*	0.021
**CF6: Leadership and Safety Culture**	9 (1 ± 0) a	16 (1.06 ± 0.25) b	6 (1.83 ± 0.98)	14 (2 ± 0.96) a,b	10 (1.8 ± 0.92)	***F*** = 5.111**	0.002
**CF7: Equipment / Technology**	7 (1 ± 0)	10 (1.1 ± 0.32)	5 (1.2 ± 0.45)	10 (1.5 ± 0.71)	6 (1.33 ± 0.52)	*F* = 1.462	0.236
**CF8: Environmental Factors**	3 (1 ± 0)	7 (1 ± 0)	4 (1 ± 0)	5 (2 ± 1.22)	5 (1.4 ± 0.89)	*F* = 1.94	0.145
**CF9: Others**	3 (1 ± 0)	6 (1 ± 0)	3 (1 ± 0)	7 (1.14 ± 0.38)	3 (1 ± 0)	*F* = 0.483	0.748
** CF10: Not Categorized**	2 (1 ± 0)	7 (1.29 ± 0.49)	4 (1 ± 0) a	3 (1 ± 0)	5 (2 ± 0.71) a	***F*** = 3.778*	0.024
**Level of Harm**							
**LH1: Death**	14 (1.21 ± 0.43) a	19 (1.37 ± 0.96) b	12 (1.42 ± 0.79) c	18 (2.28 ± 1.27)	9 (3.44 ± 2.51) a, b,c	***F*** = 6.224***	< 0.001
**LH2 No harm**	0	6 (1 ± 0)	3 (1.33 ± 0.58)	4 (2.5 ± 3)	4 (1 ± 0)	*F* = 1.01	0.42
**LH3 Permanent Harm**	1	2 (1 ± 0)	1	4 (1 ± 0)	2 (2.5 ± 2.12)	*F* = 1	0.486
**LH4 Severe temporary harm**	5 (1 ± 0)	6 (1.33 ± 0.52)	3 (1 ± 0)	3 (1.67 ± 0.58)	4 (1.25 ± 0.5)	*F* = 1.541	0.238
**Patient Safety Culture (PSC) Survey**							
**D1: Teamwork Within Units**	20 (81.28 ± 5.55) a	23 (78.53 ± 5.88) b	14 (76.59 ± 5.7)	21 (73.59 ± 5.67) a,b	11 (76.99 ± 4.08)	***F*** = 5.272***	< 0.001
**D2: Supervisor/Manager Expectations and Actions Promoting Patient Safety**	20 (63.21 ± 5)	23 (63.68 ± 7.02)	14 (62.31 ± 4.82)	21 (60.54 ± 5.75)	11 (64.10 ± 5.02)	*F* = 1.129	0.349
**D3: Organizational Learning—Continuous Improvement**	20 (80.14 ± 5.37) a	23 (79.13 ± 6.41)	14 (77.82 ± 3.46)	21 (75.34 ± 3.63) a	11 (77.46 ± 4.09)	***F*** = 2.818*	0.03
**D4: Management Support for Patient Safety**	20 (60.57 ± 8.73)	23 (60.12 ± 9.43)	14 (60.52 ± 10.09)	21 (57.68 ± 8.62)	11 (61.80 ± 5.61)	*F* = 0.511	0.728
**D5: Overall Perceptions of Patient Safety**	20 (57.71 ± 4.48)	23 (58.93 ± 5.71)	14 (57.73 ± 5.08)	21 (56.11 ± 6.09)	11 (56.90 ± 3.65)	*F* = 0.844	0.501
**D6: Feedback and Communication About Error**	20 (64.41 ± 6.78)	23 (64.89 ± 7.57)	14 (62.54 ± 6.02)	21 (60.82 ± 5.25)	11 (64.83 ± 4.53)	*F* = 1.523	0.203
** D7: Communication Openness**	20 (53.30 ± 5.64) a	23 (52.88 ± 6.25)	14 (49.59 ± 3.6)	21 (48.28 ± 6.49) a	11 (49.56 ± 5.13)	***F*** = 3.013*	0.022
**D8: Frequency of Events Reported**	20 (57.61 ± 6.56)	23 (59.22 ± 8.5)	14 (56.71 ± 6.99)	21 (57.39 ± 5.24)	11 (62.95 ± 4.71)	*F* = 1.742	0.148
**D9: Teamwork Across Units**	20 (57.62 ± 9.61)	23 (54.65 ± 9.45)	14 (52.05 ± 8.65)	21 (50.16 ± 9.35)	11 (55.03 ± 4.19)	*F* = 2.045	0.095
**D10: Staffing**	20 (30.06 ± 2.98)	23 (31.63 ± 3.75)	14 (30.88 ± 4)	21 (30.22 ± 7.09)	11 (32.27 ± 5.67)	*F* = 0.593	0.668
**D11: Handoffs and Transitions**	20 (53.08 ± 9.7) a	23 (50.81 ± 9.74)	14 (47.78 ± 7.26)	21 (44.92 ± 8.8) a	11 (49.45 ± 5.81)	***F*** = 2.516*	0.047
**D12: Nonpunitive Response to Error**	20 (23.08 ± 5.15)	23 (24.4 ± 9.09)	14 (21.95 ± 3.66)	21 (22.04 ± 8.3)	11 (24.48 ± 6)	*F* = 0.504	0.733
**Across 12 domains**	20 (56.84 ± 5.12)	23 (56.57 ± 5.53)	14 (54.70 ± 4.56)	21 (53.09 ± 5.59)	11 (56.32 ± 3.42)	*F* = 1.929	0.113
**Positive Events Reported (PER)**	20 (53.14 ± 8.28)	23 (50.78 ± 10.29)	14 (48.83 ± 8.88)	21 (48.24 ± 7.72)	11 (53.69 ± 4.71)	*F* = 1.361	0.254
**Patient Overall Safety Grade (POSG)**	20 (74.12 ± 9.13)	23 (75.17 ± 8.44)	14 (73.83 ± 6.42)	21 (74.08 ± 6)	11 (73.90 ± 6.59)	*F* = 0.104	0.981

Statistical significance markers: * *p* < 0.05; ** *p* < 0.01; *** *p* < 0.001

The significant pairwise comparison differences in means (*p* < 0.05) using Tuckey post-hoc analysis are denoted by the same letters (a,b,c,d)

### The relationship between the patient safety culture and the reported sentinel events

#### Patient safety culture domains and reported sentinel events

**Fig. 1:**
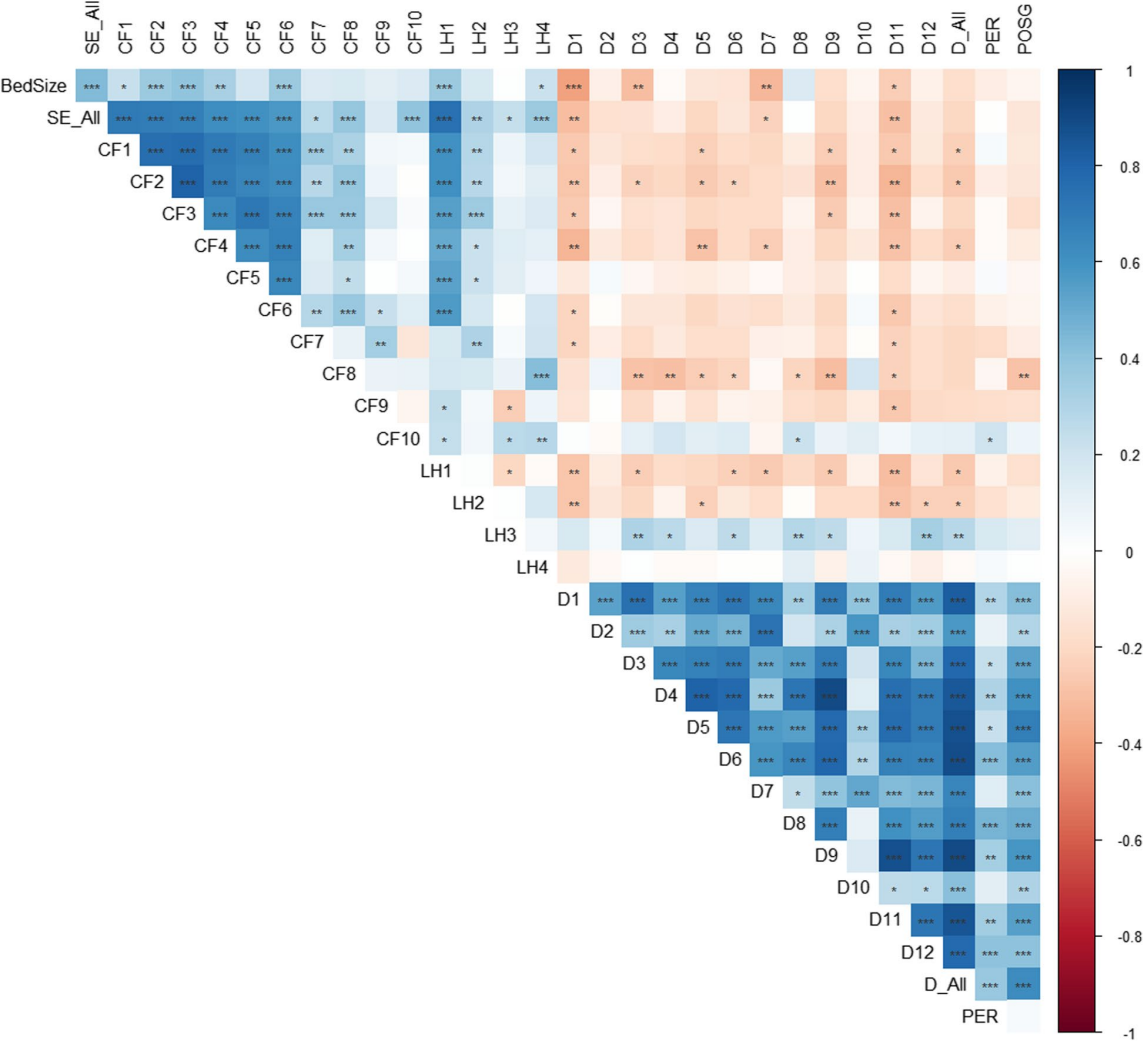
Heatmap of spearman correlations matrix. Legend: The magnitude of the r value is denoted by blue color for positive correlations and red color for negative correlations. CF1: Communication and Information; CF2: Staff Competency and Performance; CF3: Process Issues; CF4: Human Factors; CF5: Manpower Planning Issues; CF6: Leadership and Safety Culture; CF7: Equipment / Technology; CF8: Environmental Factors; CF9: Others; CF10: Not Categorized; LH1: Death; LH2: Permanent Harm; LH3: Severe temporary harm; LH4: No harm; D1: Teamwork Within Units; D2: Supervisor/Manager Expectations and Actions Promoting Patient Safety; D3: Organizational Learning—Continuous Improvement; D4: Management Support for Patient Safety; D5: Overall Perceptions of Patient Safety; D6: Feedback and Communication About Error; D7: Communication Openness; D8: Frequency of Events Reported; D9: Teamwork Across Units; D10: Staffing; D11: Handoffs and Transitions; D12: Nonpunitive Response to Error; D_All: Average percent positive across domains; PER: % Positive Events Reported (at least one event during the last 12 months); POSG: % Patient Overall Safety Grade. Statistical significance markers: * *p* < 0.05; ** *p* < 0.01; *** *p* < 0.001

Figure 1 shows the correlation matrix as a heatmap to explore the relationships between the PSC domains and the reported-SEs, CFs, and level of harm. We found negative correlations between PSC domains and reported-SEs with r coefficients ranging from –0.30 to –0.001. Higher positive scores in the PSC domains were associated significantly with lower reported-SEs rates, which are “Teamwork Within Units” (*r* = -0.30, *p* = 0.004), “Handoffs and Transitions” (*r* = -0.29, *p* = 0.006), “Communication Openness” (*r* = -0.23, *p* = 0.03). Overall higher PSC aggregated average percent positive across domains were associated with lower reported-SEs rates (*r* =  − 0.905, *p* < 0.01). By examining the relationships between reported-SEs and PSC self-reported outcome measures, POSG “overall perception of safety” and PER “positive events reported (at least one event during the last 12 months)” were not significantly related to reported-SEs. The bed size was negatively related to all of the PSC domains except “Frequency of Events Reported” domain with significant correlations between bed size and each of “Teamwork Within Units” (*r* = -0.40, *p* < 0.001), “Communication Openness” (*r* = -0.32, *p* = 0.002), "Organizational Learning—Continuous Improvement" (*r* = -0.31, *p* = 0.003), and “Handoffs and Transitions” (*r* = -0.24, *p* = 0.02). Supplementary 1 shows the spearman correlation coefficient and p-values for all of the variables.

#### Patient safety culture domains and contributory factors

We found 33 significant negative correlations (Fig. 1) between CFs of the reported-SEs and PSC measure. “Staff Competency and Performance”, “Human Factors”, and “Communication and Information”, showed significant negative associations with average percent positive across PSC domains (*r* = -0.26, -0.24, and -0.23, respectively). “Staff Competency and Performance” was correlated negatively with the following 6 PSC domains from the highest to the lowest: “Handoffs and Transitions” (*r* = -0.33, *p* = 0.002), “Teamwork Across Units” (*r* = -0.29, *p* = 0.006), “Teamwork Within Units” (*r* = -0.27, *p* = 0.010), “Overall Perceptions of Patient Safety” (*r* = -0.25* p* = 0.018), “Organizational Learning—Continuous Improvement” (*r* = -0.22, *p* = 0.037), and "Feedback and Communication About Error" (*r* = -0.22, *p* = 0.041). Furthermore, "Human Factors" was also correlated negatively with 4 PSC domains, which were "Teamwork Within Units" (*r* = -0.33, *p* = 0.001), "Overall Perceptions of Patient Safety" (*r* = -0.29, *p* = 0.007), "Handoffs and Transitions" (*r* = -0.28, *p* = 0.007), and "Communication Openness" (*r* = -0.24, *p* = 0.023). “Environmental Factors” were negatively associated with 8 measures of the PSC, notably the highest and most significant correlations among the 8 measures were "Teamwork Across Units" (*r* = -0.30, *p* = 0.004), "Management Support for Patient Safety"(*r* = -0.30, *p* = 0.005), "Patient Overall Safety Grade" (*r* = -0.29, *p* = 0.007), and "Organizational Learning—Continuous Improvement" (r = -0.28, *p* = 0.008). Remarkedly, the “Handoffs and Transitions” correlates negatively on a significant level with all the CFs apart from the “Manpower Planning Issues”. The bed size was positively correlated with five CFs, which were “Process Issues”, “Leadership and Safety Culture”, “Staff Competency and Performance”, “Human Factors”, and “Communication and Information”.

#### Patient safety culture domains and level of harm

The results (Fig. 1) showed that “Death” was negatively correlated with the following PSC domains: “Teamwork Within Units” (*r* = -0.28, *p* = 0.008), “Organizational Learning—Continuous Improvement” (*r* = -0.24, *p* = 0.02), “Feedback and Communication About Error” (*r* = -0.23, *p* = 0.03), “Communication Openness” (*r* = -0.25, *p* = 0.02), “Teamwork Across Units” (*r* = -0.26, *p* = 0.02), “Handoffs and Transitions” (*r* = -0.31, *p* = 0.003), and average percent positive across domains (*r* = -0.27, *p* = 0.01). For “Permanent Harm”, the results showed that it was negatively correlated with the following PSC domains: “Teamwork Within Units” (*r* = -0.27, *p* = 0.009), “Overall Perceptions of Patient Safety” (*r* = -0.23, *p* = 0.03), “Handoffs and Transitions” (*r* = -0.29, *p* = 0.006), “Nonpunitive Response to Error” (*r* = -0.23, *p* = 0.03), and (*r* = -0.27, *p* = 0.0.009). The bed size was positively related to the reported-SEs (*r* = 0.44, *p* < 0.001) and to “Death”(*r* = 0.37,*p* < 0.001).

#### The influence of patient safety domains on sentinel events, patient overall safety grade (POSG), and positive events reported (PER)

Multivariate regression (Table 4) was calculated to identify the influence of the 12 PSC domains on the positive percent of the number of reported-SEs (SE model), the patient overall safety grade (POSG model), and the positive events reported i.e. at least one event reported during the last 12 months (PER model). For SE Model, the 12 PSC domains explain a significant amount of variance in the number of reported-SEs which was 28.21% (F (12, 76) = 2.49, *p* = 0.008). “Handoffs and Transitions” and “Nonpunitive Response to Error” domains were statistically significant predictors of the number of SEs. “Handoffs and Transitions” domain had a greater beta with a significant negative influence on the SE (β =  − 0.672, *p* = 0.012), while “Nonpunitive Response to Error” domain positively predicted the SE (β = 0.458, *p* = 0.021). Five PSC domains accounted for 25.38% of the variance (F (5, 83) = 5.65, *p* < 0.001) in the final SE model using stepwise approach. The significant PSC predictors of the SEs were similar to the multivariate model with one more significant domain which is “Teamwork Within Units”. For POSG model, we found that the PSC domains explain a significant amount of the variance in the positive percent of the overall safety grade as a whole was 58.45% (F (12, 76) = 8.911, *p* < 0.001). The analysis showed that “Overall Perceptions of Patient Safety” and “Nonpunitive Response to Error” made a statistically significant contribution to the prediction of patient overall safety grades. “Overall Perceptions of Patient Safety” recorded a higher beta value than “Nonpunitive Response to Error”; however, “Overall Perceptions of Patient Safety” positively contributed with (β = 0.648, *p* = 0.001), and “Nonpunitive Response to Error” had a significant negative influence on the overall safety grade (β =  − 0.375, *p* = 0.013). The final POSG model using stepwise approach showed a variance of 57.65% (F (7, 81) = 15.75, *p* < 0.001) accounting for 7 PSC domains. In addition to the two significant domains that appeared in the multivariate regression, “Staffing” domain was also significant in the stepwise regression model. For the PER model, we found that the 12 PSC domains explain a significant amount of the variance in the percent positive events reported as a whole was 36.91% (F (12, 76) = 3.705, *p* < 0.001). There were no statistically significant domains in this model. The final PER model showed a variance of 33.01% (F (5, 83) = 8.18, *p* < 0.001). Unlike multivariate regression, the stepwise regression was influenced by 5 PSC domains where two of which were statistically significant predictors which are “Organizational Learning—Continuous Improvement” and “Frequency of Events Reported” (see Table 4).

**Table 4:** The multivariate linear regression and multivariate stepwise regression analyses (standardized β coefficients)

Model	Sentinel events (SE) Model		Patient overall safety grade (POSG) Model		Positive events reported (PER) Model	
**Predictors**	**Multivariate regression**	**Stepwise multivariate regression**	**Multivariate regression**	**Stepwise multivariate regression**	**Multivariate regression**	**Stepwise multivariate regression**
D1: Teamwork Within Units	-0.437	-0.341*	-0.255	-0.184	0.297	0.290
D2: Supervisor/Manager Expectations and Actions Promoting Patient Safety	0.115		-0.183	-0.148	0.007	
D3: Organizational Learning—Continuous Improvement	0.328	0.245	0.176	0.200	-0.267	-0.379*
D4: Management Support for Patient Safety	0.235	0.276	0.219	0.255	0.058	
D5: Overall Perceptions of Patient Safety	-0.244		0.648***	0.680***	-0.284	-0.201
D6: Feedback and Communication About Error	0.101		0.054		0.430	0.368
D7: Communication Openness	-0.178		0.121		-0.187	
D8: Frequency of Events Reported	-0.062		-0.039		0.351	0.438**
D9: Teamwork Across Units	0.168		0.098		-0.405	
D10: Staffing	0.061		0.211	0.215*	0.011	
D11: Handoffs and Transitions	-0.672*	-0.685***	0.023		0.297	
D12: Nonpunitive Response to Error	0.458*	0.339*	-0.375*	-0.304*	0.224	

*SE* Sentinel events, *PER* % Positive Events Reported (at least one event during the last 12 months), *POSG* % Patient Overall Safety Grade

Statistical significance markers: * *p* < 0.05; ** *p* < 0.01; *** *p* < 0.001

## Discussion

This study investigated the relationships between PSC and SEs utilizing two data sources collected on a large scale in Saudi Arabia, which are PSC survey and SEs reports. The overall percentage of positive response of PSC (58.38%) is acceptable when compared to the overall percentage (65%) of the United States in 2018 [48]. Our study identified PSC domains as areas of strength (positive areas) and areas of weakness (negative areas) in Saudi hospitals that are consistent with the other studies [9, 49–51]. The top areas of strengths in PSC domains among 366 hospitals in Saudi Arabia were “teamwork within units” and “organizational learning". The top areas of weaknesses in PSC domains were “nonpunitive response to error”, “staffing”, and “communication openness”, which may be also indicative of risky factors affecting the occurrence of SEs. On the other hand, there was a total of 231 SEs reported from 103 hospitals in Saudi Arabia. Two factors mostly contributed to these reported-SEs which are “communication and information” followed by “staff competency and performance”, consistent with previous studies in Saudi and internationally [10, 16, 52, 53]. As our objective was to investigate the relationships between the PSC and reported-SEs, merging the two datasets resulted in 89 hospitals with 195 reported-SEs that were matched by hospitals to their corresponding PSC measures. The correlation results showed a significant increase in the rates of reported-SEs among the hospitals with larger bed size. Hospitals with larger bed size might be at increased risk of more adverse levels of harm.

When we explored the relationships between PSC and the numbers of reported-SEs, our results indicated that more positive PSC is associated with lower rates of reported-SEs which was consistent with previous studies [20, 54, 55]; with 3 out of the 12 PSC domains were significantly related to lower rates of reported-SEs. These results suggested that improving positive culture in “teamwork within units”, “handoffs and transitions”, and “communication openness” were significantly associated with lower rates of reported-SEs that are consistent with Najjar et al. findings on adverse events [54]. These three PSC domains showed also lower positive culture among hospitals with bed size “301–500 beds” than in hospitals with lower bed size. Similarly, this pattern was found also with death as an outcome; expectingly, the rate gradually increased in the hospitals with larger bed size. Therefore, it is important to create a safe environment for healthcare workers to communicate openly and report mistakes without the fear of blame or punishment. Our regression analyses showed similar findings for the association between SEs and “handoffs and transitions” and “teamwork within units” domains. However, it also showed that more positive culture around “nonpunitive response to error” was influenced by higher numbers of reported-SEs. The “nonpunitive response to error” domain was one of the domains that gained the most attention as a crucial topic in research locally among Saudi Arabian hospitals and internationally [9, 16, 51, 56]. This is also evident in our national-level survey results as it was one of the lowest-scored domains among the PSC domains among 366 hospitals in Saudi Arabia. On the other hand, the association of “nonpunitive response to error” with higher numbers of reported SEs might indicate a positive culture of reporting without a fear of blame indicating a safer organization, which is a topic of debate [57–59]. This is evident in the influence of “frequency of events reported” domain on the positive events reported (PER) model as per our stepwise regression analysis. Therefore, it is suggested that caution should be taken when interpreting the results of reported incidents, such as sentinel events, and consideration of supporting data and contextual analysis is critical [59]. The overall perception of safety and the events reported (at least one event during the last 12 months) were not significantly related to a lower rate of reported-SEs.

The contributory factors explain the root causes of the reported-SEs. We found that more positive culture of "handoffs and transitions" was associated with lower rates of reported-SEs explained by 8 contributory factors, including "staff competency and performance", "process issues", "human factors", and "communication and information". This might indicate that improving handoffs and transitions among staff and healthcare professionals help in decreasing the incidence of SEs. Communication is one of the most common issues that can be triggered during and after the handoff of care. For instance, verbal communication can carry more risks during handoffs when compared to written communication [60]. The “communication and information” factors justify subfactors such as ineffective handover communication, lack of information, failure to seek support, and misunderstanding of the information. Our findings are also consistent with another study regarding communication during patient handoffs [61] which found that the language either the usage of the English language, which is not the native language, or the usage of medical terms negatively impacts communication during the handoffs. Moreover, the channel that is used during communication such as facial expression, body language, and eye contact may affect the interpretation of exchanging information [61, 62]. Furthermore, senior staff might be busy with leadership tasks, which can affect the performance and the communication of information. Additionally, the environment where this information is communicated among staff can have an impact on the quality and accuracy of the information, such as a noisy environment [60].

Among the top contributory factors in the number of significant correlations with PSC measures were “staff competency and performance” (e.g., lack of knowledge/skills/competence and inadequate supervision) and "environmental factors" (e.g., poor or inappropriate area design and noise), which indicate that these factors might also be potential areas to be improved among hospitals for a better PSC and lower reported-SEs rates. In addition to its correlation with "handoffs and transitions", “staff competency and performance” have a negative association with the domains that are linked with the teamwork aspects (“teamwork within units” and “teamwork across units”) and error prevention and positive change aspects (“organizational learning”, “overall perceptions of patient safety”, and “feedback and communication about error”. "Environmental factors" was the only contributory factor that had a negative association with the "patient overall safety grade" measure of PSC. Hayashi et al. [63] examined the relationship between PSC and the working environment and suggested that managing the work environment among healthcare workers can improve PSC. Furthermore, they found that high number of night shifts might increase the number of adverse events. Our results are in agreement with previous findings that the following factors are critical towards a more positive PSC: leadership, teamwork, evidence-based, communication, learning, just, and patient-centered [64, 65].

Such identified PSC domains and sentinel events relationships in this study might be considered risky or negative domains require further actions including mitigation actions and interventions to minimize or eliminate their negative impact on patient outcomes. Examples of interventions that hospitals can implement to improve culture and minimize unpredictable events are implementing effective reporting systems to improve “nonpunitive response to error”, appointing a safety champion for every unit to improve and conduct satisfaction surveys “staffing”, using safety briefings to improve “communication openness”, implementing Situation-Background-Assessment-Recommendation (SBAR) technique to improve “teamwork within units”, and relaying safety reports at shift change to improve “handoffs and transitions” [66]. Other examples of strategies with leadership responsibility to improve response to error, such as “Just culture”, staff supporting programs, and “good catches” [67]. “Just culture”, for example, can address two of the weakest domains which are “nonpunitive response to error” and “communication openness”. To improve communication during handoffs, the following strategies are suggested: standardization and simplification, avoiding interruptions during handoffs, limiting the use of intermediaries, using a common communication style, implementing a readback or hearback communication process, and keeping communication patient-focused. Staff and healthcare workers must feel safe during communication to speak up and actively participate during the handoffs [60]. Furthermore, the implementation of the TeamSTEPPS teamwork concept on patient safety culture among hospitals might have an impact in improving some dimensions, including “teamwork within units” and “communication openness” [54, 68].

Several areas of patient safety culture and underreporting that have been identified as weaknesses still exist among hospitals in Saudi Arabia. First, we would like to emphasize that hospital management support is critical and showed evidence of improved patient safety culture and reporting culture [65]. Second, even with the most advanced analytical methods and tools, addressing the underreporting problems requires a culture that engages the acknowledgment of errors [56]. Learning from the reported-SEs is an essential phase to improve the patient safety culture in the hospital and improve prioritizing of the recommendations [28]. 7% of the hospitals reported the process issues including stages of the task not being designed or lack of prioritization of guidelines were sub-contributors to the events; another study concluded that prioritizing recommendations with subjective criteria from the reported-SEs is an essential phase of the learning process [28, 69]. Therefore, assessing the patient safety culture and its connection to adverse events and sentinel events is a continuous learning process. Third, we believe that adopting technologies as a part of the clinical workflows and reporting process might help in providing structured information resources that can assist in more standardized reporting and better learning processes. For instance, electronic health records (EHR) nowadays can enhance the standardization of handoff communication when used effectively with greater efficiency, accountability, timeliness of communication, and data accuracy and completeness [70]. In fact, it is highly recommended for hospitals to move to data-driven approaches with transparent reporting of SEs [64] that can be facilitated by technologies. Fourth, we also would like to highlight the importance of government initiatives to improve patient safety through data-driven approaches, such as those established by the Saudi Patient Safety Center [11], which has implemented electronic-based tools to facilitate reporting adverse and sentinel events as well as participating in the patient safety culture initiatives. Fifth, our results indicated that “human factors” was among the top contributory factors, it is important to give attention to human factors during communication, such as stress and rushing to complete a task. Developing accurate and efficient healthcare systems and processes is necessary to ensure that these processes are carried out safely during situations, such as handoffs [60].

Given how patient safety culture is new in Saudi and very few studies have been published on a large scale in this area, we believe our research currently represents the first study with this context of linking patient safety culture to sentinel events and its contributory factors. One of the strengths of this study is that the study was conducted on a national level among participating hospitals in Saudi Arabia when compared to previous studies [9, 51]. Additionally, we investigated the contributory factors of the reported-SEs among different healthcare sectors (MOH, government non-MOH, and private hospitals) in Saudi Arabia. A limitation of this study is that the number of reported-SEs was relatively small in comparison to the patient safety culture survey data and merging the two datasets led to a decrease in the number of analyzed hospitals from 366 to 89, which might affect the generalizability of the results on other hospitals and other countries. This might be because the occurrence of reported-SEs is much lower than the occurrence of other adverse events in general. Moreover, data was collected nationally at the hospital level where there might be additional data that might not have been shared. Underreporting is a known issue that we hope in this study we encourage hospitals to report. This study was performed on a one-year duration, future studies can focus on change over the years in both safety culture and the rates of reported-SEs. Future studies can focus on implementing interventions and measuring their impact on PSC and the occurrence of sentinel events. Since this study focused on quantitative approaches, we suggest that future studies should focus also on qualitative approaches to analyze the relationships between patient safety culture and sentinel events. Moreover, future studies can focus on more focused contexts, such as other adverse events or specific contributory factors, and can utilize predictive approaches and machine learning to predict patient safety outcomes.

## Conclusions

At a national level in Saudi Arabia, there were limited efforts carried out to measure, unify, analyze, and generate aggregate reports and analytics that addressed patient safety culture domains and sentinel events. Communication is the most highlighted negative domain and has a negative association with the reported sentinel events. To minimize the fear of sentinel events reporting and to improve overall patient safety in Saudi Arabia a culture change is needed by promoting a blame-free culture and improving teamwork, handoffs, and communication openness. Furthermore, there was evidence that a more positive patient safety culture was associated with lower numbers of sentinel events. Lastly, we identified evidence-based areas of strengths and weakness in patient safety culture and their relationships to the contributory factors of the sentinel events that can facilitate implementing future interventions, encourage data-driven approaches to patient safety, and require attention by health organizations in Saudi Arabia and worldwide. We hope that the results of this study can help leaders and decision-makers to prioritize the efforts of improving patient safety culture among health professionals and within healthcare organizations.

## Supplementary Information

**Additional file 1:** **Appendix 1.** The percentage of reported sentinel events (SEs) and hospitals reported-SEs during 2020.

**Additional file 2: Appendix 2.** Hospital Survey on Patient Safety Culture (HSPSC) measures (descriptive calculations).

**Additional file 3: Supplementary 1.** The spearman correlation coefficient and *p*-values for all of the variables.

## Data Availability

Data that support the findings of this study are available from the Saudi Patient Safety Center (SPSC) and the authors upon request.

## Abbreviations

AHRQ: Agency for Healthcare Research and Quality
CF: Contributory factor
HSPSC: Hospital Survey on Patient Safety Culture
MOH: Ministry of Health
PER: Positive events reported
POSG: Patient overall safety grade
PSC: Patient safety culture
SE: Sentinel event

## References

[1] EEPRU - Prevalence and economic burden of medication errors in the NHS in England. https://eepru.sites.sheffield.ac.uk/projects/prevalence-and-economic-burden-of-medication-errors-in-the-nhs-in-england. Accessed 5 Sept 2022.

[2] Makary MA, Daniel M (2016). Medical error—the third leading cause of death in the US. BMJ.

[3] Elliott RA, Camacho E, Jankovic D, Sculpher MJ, Faria R (2021). Economic analysis of the prevalence and clinical and economic burden of medication error in England. BMJ Qual Saf.

[4] Tariq RA, Vashisht R, Sinha A, et al. Medication Dispensing Errors And Prevention. [Updated 2022 Jul 3]. In: StatPearls [Internet]. Treasure Island (FL): StatPearls Publishing; 2022. Available from: https://www.ncbi.nlm.nih.gov/books/NBK519065. 30085607

[5] Institute of Medicine. To Err Is Human: Building a Safer Health System. Washington, DC: The National Academies Press. 2000. 10.17226/9728. 25077248

[6] Dicuccio MH (2015). The Relationship between Patient Safety Culture and Patient Outcomes: A Systematic Review. J Patient Saf.

[7] The Joint Commission. Sentinel Events. https://www.jointcommission.org/resources/patient-safety-topics/sentinel-event/sentinel-event-policy-and-procedures/. Accessed 23 Aug 2022.

[8] Saudi Patient Safety Center. Saudi Healthcare Sentinel Event Manual. 2021. https://www.spsc.gov.sa/English/PublishingImages/Pages/RCA/SHS11.pdf. Accessed 1 July 2022.

[9] Albalawi A, Kidd L, Cowey E (2020). Factors contributing to the patient safety culture in Saudi Arabia: a systematic review. BMJ Open.

[10] Altalhi N, Alnaimi H, Chaouali M, Alahmari F, Alabdulkareem N, Alaama T (2021). Top four types of sentinel events in Saudi Arabia during the period 2016–19. Int J Qual Health Care.

[11] Saudi Patient Safety Center At a Glance. https://www.spsc.gov.sa/English/Pages/SPSC-At-A-Glance.aspx. Accessed 14 Feb 2023.

[12] Battles JB, Kaplan HS, Van der Schaaf TW, Shea CE. The attributes of medical event-reporting systems: experience with a prototype medical event-reporting system for transfusion medicine. Arch Pathol Lab Med. 1998;122(3):231–8. 9823860

[13] Anderson JG. Regional Patient Safety Initiatives: The Missing Element of Organizational Change. In A. Kushniruk & E. Borycki (Eds.), Human, Social, and Organizational Aspects of Health Information Systems. IGI Global. 2008. p 167–179. 10.4018/978-1-59904-792-8.ch010.

[14] Anderson JG, Abrahamson K (2017). Your Health Care May Kill You: Medical Errors. Stud Health Technol Inform.

[15] Hamilton EC, Pham DH, Minzenmayer AN, Austin MT, Lally KP, Tsao K (2018). Are we missing the near misses in the OR?—underreporting of safety incidents in pediatric surgery. J Surg Res.

[16] Kaud Y, O’Connor P, O’Malley R, Dunne R, Lydon S (2022). A scoping review of patient safety research carried out in Saudi Arabian hospitals. IJQHC Commun.

[17] Hatoun J, Suen W, Liu C, Shea S, Patts G, Weinberg J (2016). Elucidating Reasons for Resident Underutilization of Electronic Adverse Event Reporting. Am J Med Qual.

[18] Zohar D (2000). A group-level model of safety climate: testing the effect of group climate on microaccidents in manufacturing jobs. J Appl Psychol.

[19] Clarke S. Perceptions of organizational safety: implications for the development of safety culture. J Organiz Behav. 1999;20:185–98. 10.1002/(SICI)1099-1379(199903)20:2<185::AIDJOB892>3.0.CO;2-C.

[20] Mardon RE, Khanna K, Sorra J, Dyer N, Famolaro T (2010). Exploring Relationships Between Hospital Patient Safety Culture and Adverse Events. J Patient Saf.

[21] Alayed AS, Lööf H, Johansson UB (2014). Saudi Arabian ICU safety culture and nurses’ attitudes. Int J Health Care Qual Assur.

[22] Al-Awa B, al Mazrooa A, Rayes O, el Hati T, Devreux I, Al-Noury K (2012). Benchmarking the post-accreditation patient safety culture at King Abdulaziz University Hospital. Ann Saudi Med.

[23] al Malki A, Endacott R, Innes K (2018). Health professional perspectives of patient safety issues in intensive care units in Saudi Arabia. J Nurs Manag.

[24] Firth-Cozens J (2002). Barriers to incident reporting. Qual Saf Health Care.

[25] Read GJM, Shorrock S, Walker GH, Salmon PM (2021). State of science: evolving perspectives on “human error”. Ergonomics.

[26] Alsaeed H, Alzain N, Alquedehy R (2018). Barriers to Sentinel Events Reporting in Tertiary Hospital at Dammam, Saudi Arabia.

[27] Aljabari S, Kadhim Z. Common Barriers to Reporting Medical Errors. Scientific World Journal. 2021;2021(Article ID 6494889):8. 10.1155/2021/6494889. 10.1155/2021/6494889 PMC8211515 34220366

[28] Bos K, Dongelmans DA, Greuters S, Kamps G-J, van der Laan MJ (2020). The next step in learning from sentinel events in healthcare. BMJ Open Qual.

[29] Amaniyan S, Faldaas BO, Logan PA, Vaismoradi M (2020). Learning from Patient Safety Incidents in the Emergency Department: A Systematic Review. J Emerg Med.

[30] Alahmadi HA (2010). Assessment of patient safety culture in Saudi Arabian hospitals. Qual Saf Health Care.

[31] Hughes RG (2008). Tools and Strategies for Quality Improvement and Patient Safety.

[32] Simsekler MCE, Qazi A, Alalami MA, Ellahham S, Ozonoff A (2020). Evaluation of patient safety culture using a random forest algorithm. Reliab Eng Syst Saf.

[33] Sorra J, Gray L, Streagle S, Famolaro T, Yount N, Behm J. AHRQ Hospital Survey on Patient Safety Culture: User’s Guide. (Prepared by Westat, under Contract No. HHSA290201300003C). AHRQ Publication No. 18-0036-EF (Replaces 04-0041, 15(16)-0049-EF). Rockville: Agency for Healthcare Research and Quality. 2018. https://www.ahrq.gov/sops/quality-patientsafety/patientsafetyculture/hospital/index.html.

[34] Agency for Healthcare Research and Quality. Hospital Survey on Patient Safety Culture. https://www.ahrq.gov/sops/surveys/hospital/index.html. Accessed 21 Aug 2022.

[35] SPSC. Hospital Survey on Patient Safety Culture National Project in the Kingdom of Saudi Arabia. https://www.spsc.gov.sa/English/HSPSC/Pages/default.aspx. Accessed 23 Aug 2022.

[36] Mishra P, Pandey CM, Singh U, Gupta A, Sahu C, Keshri A (2019). Descriptive statistics and normality tests for statistical data. Ann Card Anaesth.

[37] Zhang Z. Variable selection with stepwise and best subset approaches. Ann Transl Med. 2016;4(7):136. 10.21037/atm.2016.03.35. 10.21037/atm.2016.03.35 PMC4842399 27162786

[38] R: The R Project for Statistical Computing. https://www.r-project.org/. Accessed 14 Feb 2023.

[39] What Is Tableau? | Tableau. https://www.tableau.com/why-tableau/what-is-tableau. Accessed 14 Feb 2023.

[40] SPSS Statistics | IBM. https://www.ibm.com/products/spss-statistics. Accessed 14 Feb 2023.

[41] Microsoft Excel Spreadsheet Software | Microsoft 365. https://www.microsoft.com/en-us/microsoft-365/excel. Accessed 14 Feb 2023.

[42] Huntington-Klein N. Variable Table for Variable Documentation [R package vtable version 1.3.4]. 2022. https://nickch-k.github.io/vtable/.

[43] TukeyHSD function - RDocumentation. https://www.rdocumentation.org/packages/stats/versions/3.6.2/topics/TukeyHSD. Accessed 15 Sep 2022.

[44] cor function - RDocumentation. https://www.rdocumentation.org/packages/stats/versions/3.6.2/topics/cor. Accessed 15 Sep 2022.

[45] Visualization of a Correlation Matrix [R package corrplot version 0.92]. 2021. https://cran.r-project.org/web/packages/corrplot/index.html. Accessed 15 Sep 2022.

[46] Kassambara A. Visualization of a Correlation Matrix using “ggplot2” [R package ggcorrplot version 0.1.3]. 2019. https://cran.r-project.org/web/packages/ggcorrplot/index.html. Accessed 15 Sep 2022.

[47] R: Choose a model by AIC in a Stepwise Algorithm. https://stat.ethz.ch/R-manual/R-devel/library/MASS/html/stepAIC.html. Accessed 10 Feb 2023.

[48] Famolaro T, Yount ND, Hare R, Thornton S, Kristi Meadows, Fan L, et al. Hospital Survey on Patient Safety Culture 2018 Database Report. Rockville: Agency for Healthcare Research and Quality; 2018.

[49] Alahmadi HA (2010). Assessment of patient safety culture in Saudi Arabian hospitals. BMJ Qual Saf.

[50] Huang H, Xiao L, Chen Z, Cao S, Zheng S, Zhao Q, et al. A National Study of Patient Safety Culture and Patient Safety Goal in Chinese Hospitals. J Patient Saf. 2022;18(8):e1167–e1173. 10.1097/PTS.0000000000001045. 10.1097/PTS.0000000000001045 PMC9698193 35617631

[51] Elmontsri M, Almashrafi A, Banarsee R, Majeed A (2017). Status of patient safety culture in Arab countries: a systematic review. BMJ Open.

[52] Methangkool E, Tollinche L, Sparling J, Agarwala AV (2019). Communication: Is There a Standard Handover Technique to Transfer Patient Care?. Int Anesthesiol Clin.

[53] Steelman VM, Shaw C, Shine L, Hardy-Fairbanks AJ (2019). Unintentionally Retained Foreign Objects: A Descriptive Study of 308 Sentinel Events and Contributing Factors. Jt Comm J Qual Patient Saf.

[54] Najjar S, Nafouri N, Vanhaecht K, Euwema M (2015). The relationship between patient safety culture and adverse events: a study in palestinian hospitals. Safety in Health.

[55] Muething SE, Goudie A, Schoettker PJ, Donnelly LF, Goodfriend MA, Bracke TM (2012). Quality Improvement Initiative to Reduce Serious Safety Events and Improve Patient Safety Culture. Pediatrics.

[56] Nieva VF, Sorra J (2003). Safety culture assessment: a tool for improving patient safety in healthcare organizations. BMJ Qual Saf.

[57] González-Formoso C, Clavería A, Fernández-Domínguez MJ, Lago-Deibe FL, Hermida-Rial L, Rial A (2019). Effectiveness of an educational intervention to improve the safety culture in primary care: A randomized trial. BMC Fam Pract.

[58] Howell AM, Burns EM, Bouras G, Donaldson LJ, Athanasiou T, Darzi A (2015). Can Patient Safety Incident Reports Be Used to Compare Hospital Safety? Results from a Quantitative Analysis of the English National Reporting and Learning System Data. PLoS ONE.

[59] Hutchinson A, Young TA, Cooper KL, McIntosh A, Karnon JD, Scobie S (2009). Trends in healthcare incident reporting and relationship to safety and quality data in acute hospitals: results from the National Reporting and Learning System. BMJ Qual Saf.

[60] Streitenberger K, Breen-Reid K, Harris C (2006). Handoffs in Care—Can We Make Them Safer?. Pediatr Clin North Am.

[61] Solet DJ, Norvell JM, Rutan GH, Frankel RM (2005). Lost in translation: challenges and opportunities in physician-to-physician communication during patient handoffs. Acad Med.

[62] Baron RA, Byrne D. Social psychology: Understanding human interaction, 5th ed. - PsycNET. 1987. https://psycnet.apa.org/record/1986-98707-000. Accessed 6 Sep 2022.

[63] Hayashi R, Fujita S, Iida S, Nagai Y, Shimamori Y, Hasegawa T (2020). Relationship of patient safety culture with factors influencing working environment such as working hours, the number of night shifts, and the number of days off among healthcare workers in Japan: A cross-sectional study. BMC Health Serv Res.

[64] Sammer CE, Lykens K, Singh KP, Mains DA, Lackan NA (2010). What is Patient Safety Culture? A Review of the Literature. J Nurs Scholarsh.

[65] Theodosios S. The development of patient safety culture. Health Science Journal. 6:0–0. https://www.itmedicalteam.pl/articles/the-development-of-patient-safety-culture-105599.html.

[66] Adams-Pizarro I, Walker ZA, Robinson J, et al. Using the AHRQ Hospital Survey on Patient Safety Culture as an Intervention Tool for Regional Clinical Improvement Collaboratives. In: Henriksen K, Battles JB, Keyes MA, et al., editors. Advances in Patient Safety: New Directions and Alternative Approaches (Vol. 2: Culture and Redesign). Rockville: Agency for Healthcare Research and Quality (US); 2008. Available from: https://www.ncbi.nlm.nih.gov/books/NBK43728/. 21249911

[67] Fencl JL, Willoughby C, Jackson K (2021). Just Culture: The Foundation of Staff Safety in the Perioperative Environment. AORN J.

[68] Staines A, Lécureux E, Rubin P, Baralon C, Farin A (2020). Impact of TeamSTEPPS on patient safety culture in a Swiss maternity ward. Int J Qual Health Care.

[69] Hibbert PD, Thomas MJW, Deakin A, Runciman WB, Braithwaite J, Lomax S (2018). Are root cause analyses recommendations effective and sustainable? An observational study. Int J Qual Health Care.

[70] Wheeler KK (2015). Effective handoff communication. Nurs Crit Care (Ambler).

