# Supplementary material for: The relationships between patient safety culture and sentinel events among hospitals in Saudi Arabia: a national descriptive study
Source: BMC Health Serv Res. 2023 Mar 18;23:270. doi: 10.1186/s12913-023-09205-0 (PMC10024850; doi:10.1186/s12913-023-09205-0)
Supplement: Supplementary file 1 — Additional file 1: Appendix 1. The percentage of reported sentinel events (SEs) and hospitals reported-SEs during 2020. [file 12913_2023_9205_MOESM1_ESM.docx]

**Appendix1:** The percentage of reported sentinel events (SEs) and hospitals reported-SEs during 2020

| **Item** | **Number of reported SEs (%)**  **(n=231)** | **Number of hospitals reported SEs (%)**  **(n=103)** |
| --- | --- | --- |
| **Region** | | |
| Riyadh | 73 (31.6%) | 22 (21.36%) |
| Makkah | 53 (22.94%) | 26 (25.24%) |
| Eastern | 42 (18.18%) | 16 (15.53%) |
| Assir | 16 (6.93%) | 10 (9.71%) |
| Madinah | 13 (5.63%) | 7 (6.8%) |
| Tabuk | 7 (3.03%) | 5 (4.85%) |
| Al Jawf | 6 (2.6%) | 2 (1.94%) |
| Hail | 6 (2.6%) | 3 (2.91%) |
| Jizan | 4 (1.73%) | 2 (1.94%) |
| Qassim | 4 (1.73%) | 3 (2.91%) |
| Najran | 4 (1.73%) | 4 (3.88%) |
| Northern Borders | 2 (0.87%) | 2 (1.94%) |
| Al Baha | 1 (0.43%) | 1 (0.97%) |
| **Bed Capacity** | | |
| 50-100 | 26 (11.26%) | 23 (22.33%) |
| 101-200 | 44 (19.05%) | 25 (24.27%) |
| 201-300 | 32 (13.85%) | 17 (16.5%) |
| 301-500 | 67 (29%) | 25 (24.27%) |
| 501+ | 62 (26.84%) | 13 (12.62%) |
| **Level of Harm** | | |
| Death | 155 (67.1%) | 81 (78.64%) |
| Severe temporary harm | 32 (13.85%) | 25 (24.27%) |
| Permanent Harm | 19 (8.23%) | 15 (14.56%) |
| No harm | 25 (10.82%) | 18 (17.48%) |
| **Who was affected?** | | |
| Patient | 218 (94.37%) | 101 (98.06%) |
| Organization | 9 (3.90%) | 4 (3.88%) |
| Staff | 4 (1.73%) | 4 (3.88%) |
| **Event Location (Number of reported SEs > 1)** | | |
| Intensive care unit (any type) | 49 (21.21%) | 39 (37.86%) |
| Operation room (OR) | 41 (17.75%) | 30 (29.13%) |
| Obstetrics | 38 (16.45%) | 26 (25.24%) |
| Emergency department | 34 (14.72%) | 27 (26.21%) |
| Surgery | 13 (5.63%) | 11 (10.68%) |
| Medicine (non-surgical) | 7 (3.03%) | 7 (6.8%) |
| Coronary Care Unit (CCU) | 6 (2.6%) | 5 (4.85%) |
| Radiology | 5 (2.16%) | 5 (4.85%) |
| Neonatal intensive care unit (NICU) | 5 (2.16%) | 5 (4.85%) |
| Psychiatry/mental health | 4 (1.73%) | 4 (3.88%) |
| Isolation ward | 4 (1.73%) | 4 (3.88%) |
| Outpatient Department (OPD) | 3 (1.3%) | 2 (1.94%) |
| Oncology, Hematology | 3 (1.3%) | 3 (2.91%) |
| Primary Health Care (PHC) | 2 (0.87%) | 2 (1.94%) |
| Pediatrics | 2 (0.87%) | 2 (1.94%) |
