# Supplementary material for: The relationships between patient safety culture and sentinel events among hospitals in Saudi Arabia: a national descriptive study
Source: BMC Health Serv Res. 2023 Mar 18;23:270. doi: 10.1186/s12913-023-09205-0 (PMC10024850; doi:10.1186/s12913-023-09205-0)
Supplement: Supplementary file 2 — Additional file 2: Appendix 2. Hospital Survey on Patient Safety Culture (HSPSC) measures (descriptive calculations). [file 12913_2023_9205_MOESM2_ESM.docx]

**Appendix 2: HSPSC measures (descriptive calculations)**

| **Item** | **Number of Respondents (n=124,891)** | **Number of Hospitals**  **(n=366)** |
| --- | --- | --- |
| **Region** | | |
| Riyadh | 31,871 (25.52%) | 66 (18.03%) |
| Eastern | 14,689 (11.76%) | 35 (9.56%) |
| Jeddah | 13,590 (10.88%) | 30 (8.2%) |
| Assir | 7,128 (5.71%) | 26 (7.1%) |
| Qassim | 6,321 (5.06%) | 25 (6.83%) |
| Madinah | 5,290 (4.24%) | 22 (6.01%) |
| Taif | 5,091 (4.08%) | 20 (5.46%) |
| Jizan | 2,978 (2.38%) | 19 (5.19%) |
| Hail | 2,982 (2.39%) | 17 (4.64%) |
| Makkah | 11,274 (9.03%) | 16 (4.37%) |
| Tabuk | 5,111 (4.09%) | 14 (3.83%) |
| Najran | 4,086 (3.27%) | 13 (3.55%) |
| Al-Ahsa | 3,887 (3.11%) | 12 (3.28%) |
| **Bed Capacity** | | |
| 50-100 | 25,403 (20.34%) | 199 (54.37%) |
| 101-200 | 20,574 (16.47%) | 71 (19.4%) |
| 201-300 | 22,468 (17.99%) | 46 (12.57%) |
| 301-500 | 28,622 (22.92%) | 35 (9.56%) |
| 501+ | 27,824 (22.28%) | 15 (4.1%) |
| **Work Area** | | |
| Emergency Department | 10,979 (8.79%) | 349 (95.36%) |
| Intensive care unit (any type) | 10,523 (8.43%) | 260 (71.04%) |
| Outpatient Department | 7,537 (6.03%) | 347 (94.81%) |
| Surgery | 7,516 (6.02%) | 312 (85.25%) |
| Laboratory | 6,974 (5.58%) | 348 (95.08%) |
| Medicine (non-surgical) | 6,900 (5.52%) | 341 (93.17%) |
| Obstetrics | 6,867 (5.5%) | 287 (78.42%) |
| Pharmacy | 6,108 (4.89%) | 354 (96.72%) |
| Pediatrics | 6,078 (4.87%) | 305 (83.33%) |
| Many different No specific unit | 5,645 (4.52%) | 349 (95.36%) |
| Radiology | 5,635 (4.51%) | 352 (96.17%) |
| Operation Room (OR) | 3,643 (2.92%) | 298 (81.42%) |
| Other | 22,566 (18.07%) | 363 (99.18%) |
| **Staff Position** | | |
| Registered Nurse | 42,485 (34.02%) | 365 (99.73%) |
| Attending/Staff Physician | 13,544 (10.84%) | 363 (99.18%) |
| Nurse Practitioner / Technician | 12,545 (10.04%) | 363 (99.18%) |
| Technician (e.g., EKG, Lab, Radiology) | 9,287 (7.44%) | 354 (96.72%) |
| Resident Physician/Physician in Training | 7,336 (5.87%) | 354 (96.72%) |
| Pharmacist / Pharmacy Technician | 6,199 (4.96%) | 356 (97.27%) |
| Duty charge / Supervisor /Manager / Director / Senior Leader / Executive | 5,953 (4.77%) | 360 (98.36%) |
| Physical, Occupational, or Speech Therapist | 2,438 (1.95%) | 278 (75.96%) |
| Physician Assistant | 1,913 (1.53%) | 290 (79.23%) |
| Dietician | 1,835 (1.47%) | 294 (80.33%) |
| Patient Care Asst/Hospital Aide/Care Partner | 1,651 (1.32%) | 243 (66.39%) |
| Unit Assistant | 1,463 (1.17%) | 236 (64.48%) |
| Other | 14,535 (11.64%) | 363 (99.18%) |
| **Working Hours per Week** | | |
| 40 to 59 hours per week | 92,579 (74.13%) | 366 (100%) |
| 60 to 79 hours per week | 14,033 (11.24%) | 359 (98.09%) |
| 20 to 39 hours per week | 10,706 (8.57%) | 363 (99.18%) |
| 80 to 99 hours per week | 3,044 (2.44%) | 328 (89.62%) |
| Less than 20 hours per week | 2,508 (2.01%) | 315 (86.07%) |
| 100 hours per week or more | 2,021 (1.62%) | 301 (82.24%) |
| **Language** | | |
| Arabic | 67,501 (54.05%) | 366 (100%) |
| English | 57,390 (45.95%) | 358 (97.81%) |
| **Interaction with patient** | | |
| Yes | 106,278 (85.1%) | 366 (100%) |
| No | 18,613 (14.9%) | 364 (99.45%) |
